# NAD+ Precursors and Intestinal Inflammation: Therapeutic Insights Involving Gut Microbiota

**DOI:** 10.3390/nu15132992

**Published:** 2023-06-30

**Authors:** Julia Niño-Narvión, Marina Idalia Rojo-López, Patricia Martinez-Santos, Joana Rossell, Antonio J. Ruiz-Alcaraz, Núria Alonso, Bruno Ramos-Molina, Didac Mauricio, Josep Julve

**Affiliations:** 1 Institut d’Investigació Biomèdica Sant Pau (IIB Sant Pau), 08041 Barcelona, Spain; julianng99@gmail.com (J.N.-N.); nut.marina.rojo.l@gmail.com (M.I.R.-L.); pat.martinez@gmail.com (P.M.-S.); jrossell@santpau.cat (J.R.); 2 Grupo de Obesidad y Metabolismo, Instituto Murciano de Investigación Biosanitaria (IMIB), 30120 Murcia, Spain; brunoramosmolina@gmail.com; 3 Departamento de Bioquímica y Biología Molecular B e Inmunología, Facultad de Medicina, Universidad de Murcia (UMU), 30120 Murcia, Spain; ajruiz@um.es; 4 CIBER de Diabetes y Enfermedades Metabólicas Asociadas, Instituto de Salud Carlos III, 08041 Barcelona, Spain; 5 Department of Endocrinology & Nutrition, Hospital de la Santa Creu i Sant Pau, 08041 Barcelona, Spain; 6 Department of Endocrinology & Nutrition, Hospital Universitari Germans Trias I Pujol, 08916 Badalona, Spain; nalonso32416@yahoo.es; 7 Faculty of Medicine, University of Vic/Central University of Catalonia (UVIC/UCC), 08500 Vic, Spain

**Keywords:** diabetes mellitus, niacin, aging, nicotinamide, metabolomics, biomarker, therapy, intestinal bowel disease, colitis, pellagra

## Abstract

The oxidized form of nicotinamide adenine dinucleotide (NAD+) is a critical metabolite for living cells. NAD+ may act either as a cofactor for many cellular reactions as well as a coenzyme for different NAD+-consuming enzymes involved in the physiological homeostasis of different organs and systems. In mammals, NAD+ is synthesized from either tryptophan or other vitamin B3 intermediates that act as NAD+ precursors. Recent research suggests that NAD+ precursors play a crucial role in maintaining the integrity of the gut barrier. Indeed, its deficiency has been associated with enhanced gut inflammation and leakage, and dysbiosis. Conversely, NAD+-increasing therapies may confer protection against intestinal inflammation in experimental conditions and human patients, with accumulating evidence indicating that such favorable effects could be, at least in part, mediated by concomitant changes in the composition of intestinal microbiota. However, the mechanisms by which NAD+-based treatments affect the microbiota are still poorly understood. In this context, we have focused specifically on the impact of NAD+ deficiency on intestinal inflammation and dysbiosis in animal and human models. We have further explored the relationship between NAD+ and improved host intestinal metabolism and immunity and the composition of microbiota in vivo. Overall, this comprehensive review aims to provide a new perspective on the effect of NAD+-increasing strategies on host intestinal physiology.

## 1. Background

Vitamin B3 exists in different molecular forms in living beings [1] with nicotinamide (NAM) and nicotinic acid (NA) being the main members. Although the term NA has been replaced by niacin to avoid any confusion with nicotine [2], both terms are indistinguishably used in the literature. Niacin specifically refers to the acid form of vitamin B3, but it is often erroneously used as a synonym for vitamin B3; however, the amide form of vitamin B3, also defined as NAM (also referred to as niacinamide in analogy to niacin), is also a main representative of vitamin B3.

In mammals, tryptophan is the only substrate available for the endogenous synthesis of nicotinamide adenine dinucleotide (NAD+). NAD+ acts as a cofactor in key cellular metabolic reactions, but is also an essential cosubstrate for different NAD+-consuming enzymes, such as protein deacetylases, and thereby influences a wide range of key cellular processes [1]. The importance of this amino acid as an endogenous source of NAD+ in human physiology was first revealed in conditions of NAD+ deficiency syndrome that commonly develop in cases of extreme nutritional deficiency [3]. Pellagra is a systemic disease typically characterized by adverse dermatological, intestinal, and neurological outcomes in affected subjects. Remarkably, these same symptoms were also seen in subjects with a genetic disorder known as Hartnup disease, which results from mutations in the SLC6A19 gene that encodes a neutral amino acid transporter, leading to reduced absorption of tryptophan [4,5]. Subjects bearing this gene mutation exhibit all the symptoms of pellagra. Pellagra is now a treatable condition; however, this was not the case before the discovery of NA (the so-called niacin) in the 1920s, a period in which pellagra was endemic in the United States [6]. Likewise, the life expectancy of subjects with Hartnup disease is also shorter if left untreated with NA. Indeed, most of the symptoms seen in subjects with Hartnup disease can be remitted using milligram quantities of NA as a NAD+ precursor [7]. Beyond its role as a NAD+ precursor, NA can also directly bind to a G-protein-coupled receptor, termed GPR109a, which has been identified as the mediator of its anti-inflammatory effect on activated macrophages [8].

**Figure 1:**
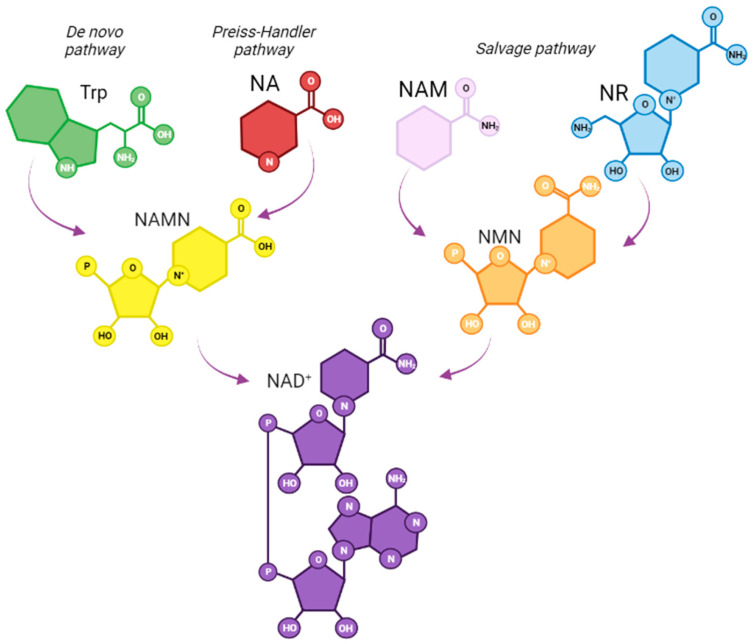
NAD+ biosynthesis in mammals. The de novo pathway uses tryptophan as precursor and involves a series of enzymatic conversions to nicotinic acid mononucleotide (NAMN) to generate NAD+. The salvage pathway involves the synthesis of NAD+ from its precursors, including nicotinic acid (NA), which leads to the synthesis of a NAMN, and it is further transformed into NAD+. nicotinamide (NAM) and nicotinamide riboside (NR) can converge into the synthesis of nicotinamide mononucleotide (NMN), another common intermediate for NAD+ synthesis.

Apart from NA, other recognized physiological sources of the oxidized form of NAD+, including NAM and other NAM derivatives, such as nicotinamide mononucleotide (NMN) and nicotinamide riboside (NR), may also be used as physiological NAD+ sources [1] (Figure 1).

Accumulating research links altered gut function to NAD+ deficiency [9,10,11]. Some chronic intestinal diseases with an altered immunomodulatory basis (i.e., Crohn’s disease, inflammatory bowel disease (IBD), etc.) have been associated with classical NAD+ deficiency [9,12], suggesting a bidirectional relationship between NAD+ depletion and intestinal inflammation.

Intestinal NAD+ biology plays an important role in intestinal homeostasis, and the administration of NMN, one of the NAD+ precursors, increases intestinal NAD+ biosynthesis and positively influences gut physiology in vivo [13] Furthermore, NAD+ precursors have been described as potent anti-inflammatory agents. In particular, intestinal inflammation may be rescued using NAD+-increasing approaches [9,14]. This beneficial effect may be elicited directly, as is the case with NA, through a direct interaction with the GPR109a receptor, which inhibits the immune activation of resident macrophages and protects against further inflammatory cell infiltration, or indirectly after their conversion into NAD+. Furthermore, signaling pathways involving the NAD+-dependent protein deacetylase sirtuin 1 (SIRT1) have been implicated in inflammation [15].

Apart from NA-mediated effects, other NAD+ precursors also exert anti-inflammatory effects by preventing inflammation in tissue-resident immune cells, such as macrophages, which are ultimately involved in the resolution of tissue inflammation [16]. Moreover, some of those precursors can also promote differentiation to macrophages with attenuated proinflammatory characteristics [16]. However, whether these effects could also be favorably influencing gastrointestinal inflammation and function has been poorly addressed.

Several inflammatory conditions affecting the intestinal wall can be attributed to alterations in the intestinal microbiota or a disrupted interplay between the intestinal microbiota and the intestines. The intestinal microbiota also plays a role in modulating NAD+ bioavailability in vivo. Thus, alterations in the intestinal microbiota can lead to functional vitamin B3 deficiency [17]. Moreover, the intestinal microbiota profoundly impacts host NAD+ metabolism by promoting host NAD+ synthesis [10]. Conversely, recent research supports the notion that vitamin B3-related supplements can have a beneficial effect on intestinal permeability [18] and inflammation [19,20,21] by modulating the composition of intestinal microbiota.

Current evidence supports the notion that NAD+ deficiency may promote intestinal inflammation and leakage, negatively influencing intestinal microbiota [22,23] and, overall host physiology; however, only a few studies have addressed the favorable influence of intestinal microbiota in response to NAD+-increasing strategies. Since NAD+ depletion is linked to inflamed intestinal tissue [9], it could be suggested that NAD+ reduction could even be exacerbated in pellagra-like conditions, leading to enhanced tissue-specific inflammation due to concomitant tissue NAD+ reductions, in a kind of vicious cycle. Under these physiopathological conditions, NAD+ and its precursors may have a therapeutic potential for the treatment of different pathologies, such as autoimmune diseases or inflammatory diseases with some having a lower risk of serious adverse drug outcomes [24].

NAD+ depletion in tissues is also a commonly observed feature in many human disorders associated with metabolic abnormalities [25]. These disorders are also linked to intestinal inflammation, and are further accompanied by altered gut microbiota [13,26,27], making NAD+ depletion an emerging therapeutic target for future therapies. However, the influence of NAD+ supplementation on the relative composition of intestinal microbiota and its derived metabolic consequences is still poorly understood. Nevertheless, accumulating evidence suggests that some NAD+ precursors may protect against intestinal inflammation with this linked to favorable changes in intestinal microbiota. Therefore, in this review, we aim to discuss the impact of NAD+ deficiency and NAD+-increasing approaches on intestinal inflammation and microbiota, and their impact on host physiology and immunity.

## 2. Intestinal Inflammation and Dysbiosis in NAD+-Deficient States

In the context of intestinal inflammation syndromes, states of NAD+ deficiency share common clinical features, including signs of intestinal inflammation and impaired gastrointestinal function (i.e., IBD) [9,11]. Several factors may contribute to reduced NAD+ content in target tissues, which can result, at least in part, in adverse outcomes in different physiological systems.

NAD+ content deficiency can be caused by decreased bioavailability, reduced use, or both. As such, it may be influenced by either modifiable factors, i.e., diet, alcoholism, or non-modifiable factors, i.e., age, certain disease conditions, or both.

Some findings suggest that NAD+ deficiency, as seen in pellagra and other conditions of reduced NAD+ bioavailability, can lead to intestinal inflammation [1,11]. Moreover, the occurrence of dysbiosis, which can be defined as an imbalance between beneficial commensal versus opportunistic bacteria, is also a common feature of intestinal inflammation. However, the direction of the sequence of events is unclear; thus, it is not yet known whether intestinal dysbiosis is causally or casually related to intestinal inflammation or to persistent low-grade inflammation associated with cardiometabolic diseases, such as obesity or diabetes [28,29]. In addition, NAD+ levels are also affected by aging [30]; however, its relationship with impaired intestinal function or dysbiosis has not been studied yet.

### 2.1. Modifiable Factors

#### 2.1.1. Pellagra, as an Example of Nutritional Deficiency

Dietary deprivation of NAD+ precursors result in serious health problems in mammals [31]. Pellagra is the term used to define this clinical syndrome that develops as a result of classical NAD+ deficiency. Its physiopathology is characterized by a tetrad of “D symptoms”: dermatitis, diarrhea, dementia, and possibly death as the ultimate outcome [22]. In general, all these symptoms are rescued after NAD+ precursor-based therapy, which is widely used for the treatment of pellagra [6].

Similar to pellagra, the most common form of irritable bowel syndrome typically involves diarrhea as a primary symptom. Indeed, the gut is one of the compartments primarily affected by NAD+ deficiency. In line with this, mild to moderate damage to the jejunal mucosa (grade 3 histological abnormality) has been reported in some of the affected subjects [23].

In developed countries, primary malnutrition, which is considered as the main cause of pellagra, is almost non-existent. However, chronic alcoholism, malabsorption syndrome, anorexia nervosa, and drug-induced NAD+ deficiency (i.e., chemotherapy) are among the primary culprits responsible for its onset [22].

Intake of NAD+ precursors and intestinal microbiota are mutually regulated [31,32]. Although diet is a critical requirement for the availability of NAD+ precursors, the contribution of microbiota to the intestinal production of these molecules is becoming increasingly recognized [33]. In this regard, some bacteria can convert host-derived NAM into NA, which is used for NAD+ synthesis [32]. Indeed, microbiota may maintain serum NA concentrations, even in the absence of dietary intake. It has been described that a low-vitamin B3 diet can alter the gut microbiota, which appears to have a key role in the development of pellagra [31]. Additionally, gut microbiota appears to be a therapeutic target for pellagra-like symptoms [31]. In line with this concept, the use of broad-spectrum antibiotics can cause alterations in the microbiome of those individuals who already have a poor diet, which can lead to the clearance of symbiotic microorganisms, thus triggering clinical vitamin B3 deficiency [34]. In animal models (mainly mice), it has been found that the gut microbiota has a significant impact on the onset of pellagra, and that pellagra resulting from a diet deficient in vitamin B3 intermediates may also alter gut microbiota [31].

#### 2.1.2. Chronic Alcoholism

The chronic intake of alcohol can lead to the development of secondary pellagra as a result of malnutrition, which is also linked to NAD+ deficiency [35]. Moreover, alcohol intake can worsen the effects of nutritional deficiencies and produce metabolic effects that include changes in the conversion of tryptophan to NA, zinc deficiency, disruption of heme biosynthesis, and alterations in the activity of glutamate and GABA neurons [36].

NA serves as the precursor to NAD+, a vital component in energy metabolism that plays a critical role in clearing ethanol/acetaldehyde [37]. Due to the presence of alcohol dehydrogenase in all visceral organs of the body, there is a reduction in NAD+ levels throughout the system during episodes of binge drinking, which can result in multi-organ injury [38]. Furthermore, when blood alcohol levels are high, NAD+ is transformed into its reduced state (NADH), which then becomes unavailable to function as a coenzyme in the deacetylation of molecules through the SIRT deacetylases [38].

Additionally, long-term alcohol abuse leads to a rise in intestinal permeability and produces modifications to the composition and functioning of the intestinal microbiota [39]. The intestinal damage due to alcohol consumption includes cell death, mucosal ulcers, erosion, and loss of epithelium, as well as oxidative stress as a result of the alcohol metabolism [39]. Alcohol and its metabolites affect the tight junction complex that joins cells, disrupting its barrier function and causing paracellular permeability. This effect is caused by the redistribution of proteins and altered expression of tight junction proteins [39,40], creating an opening for bacterial gut metabolites to enter the bloodstream. These metabolites are identified by immune cells that travel through the bloodstream; as a result, these immune cells produce and release pro-inflammatory cytokines.

#### 2.1.3. Chemotherapy

NAD+ depletion is a frequent feature in cancer patients, and chemotherapy can induce pellagra [41,42]. Such NAD+ depletion mechanism is likely to involve DNA-damaging chemotherapeutics stimulating PARP1 activity, which can be further consuming cellular NAD+ content to generate poly (ADP)ribose. Based on rat studies, pharmacological doses of NAD+ precursors may be beneficial to treat the DNA damage caused by agents used in chemotherapies [43]. In this regard, the pharmacological administration of NA or NAM increased the latency of the ethylnitrosourea-induced morbidity in treated rats. However, more experiments are needed to determine whether NAD precursor supplementation would help or worsen the situation in the context of an indoleamine 2,3-dioxygenase (IDO) inhibitor chemotherapeutic strategy.

Chemotherapy can negatively influence gut inflammation and microbiota [44,45], which is, in turn, closely related to the occurrence of an adverse intestinal syndrome, which has been defined as chemotherapy-induced intestinal mucositis [44,45]. As such, chemotherapies may seriously worsen patients’ quality of life and also their adherence to anticancer therapies. Although the beneficial effect of NAD+-increasing therapies in chemotherapy-induced gut inflammation and dysbiosis is still poorly addressed, they might be considered as a potential new therapeutic approach to alleviate and treat chemotherapy-induced intestinal mucositis.

### 2.2. Non-Modifiable Factors

#### 2.2.1. Aging

Aging can be defined as a process in which a gradual accumulation of cellular damage occurs. NAD+ levels are negatively influenced by metabolic stress and age-related diseases in preclinical models [46,47,48,49]. Such NAD+ depletion could be at least in part explained by an increased activity of poly(ADP) ribosyl polymerases (PARP), a DNA repair enzyme, which is upregulated upon aging [50]. Conversely, increasing NAD+ levels is believed to reduce the aging process and age-related diseases [51]. It is noteworthy that an increase in dysbiosis occurs at a similar rate to the aging process [52].

NAD+ depletion in different tissues promotes “inflammaging” [53,54,55,56], a term that describes a state of age-related chronic systemic sub-optimal inflammation. Inflammaging is characterized by the accumulation of several features, including altered microbiota composition, increased intestinal permeability, cellular senescence, activation of the NOD-, LRR- and pyrin domain-containing protein 3 (NLRP3) inflammasome, immune cell dysregulation, and oxidative stress resulting from dysfunctional mitochondria, among others [56].

The importance of NAD+ content in intestinal physiology has been revealed by a recent study [57], in which the pharmacological inhibition of nicotinamide phosphoribosyltransferase (NAMPT), the rate-limiting enzyme for NAD+ synthesis, significantly decreased colonic NAD content, and led to gastrointestinal dysfunction in young mice. Conversely, NAD+ replenishment protects against colon degeneration in aged mice by improving defecation. However, the impact on inflammation was not further explored; therefore, further research is required in this field.

The aging process naturally leads to changes in the intestinal composition of microbiota [52] and hence impacts the production rate of related metabolites [51]. Remarkably, intestinal dysbiosis can trigger “inflammaging” [58] with certain bacteria being associated with unhealthy ageing. *Bacteroidaceae*, *Ruminococcaceae* and *Lachnospiracae* are the least abundant bacterial families among the elderly [57]. Such a bacterial profile has been linked to the appearance of age-related degenerative diseases, unhealthy aging, and reduced longevity [59].

#### 2.2.2. Sex

Many major autoimmune diseases, including intestinal conditions, often display sexual dimorphism as a distinctive trait; women typically experience a higher incidence and/or severity of most of these diseases. Hormones and the microbiota may interact to modify the disease course, as evidenced by recent research [60]. This is not a recent phenomenon, as historical records of pellagra epidemics of the 1920s revealed that over 70% of the cases were women. Remarkably, these values of relative incidence are similar to the current frequency of autoimmune diseases observed in women [61]. Recent data suggests that the host metabolome is significantly influenced by the sex of the person [62]. In the specific case of NAD+ metabolome, some studies have shown that the plasma NAD+/NADH redox ratio is significantly higher in women than men [63], with differences in female hormones partly accounting for this disparity. Similarly, in a recent publication, the analysis of the fecal microbiota in men, women, and castrated men found that sex hormones have a greater influence on microbiota composition than factors associated with the X chromosome [60].

Recent data also indicate that NAD+ levels follow clock-controlled, rhythmic patterns that are also sex-specific [64], thus highlighting the importance of chronobiological determinants of NAD+-based therapies according to sex.

#### 2.2.3. Immunomodulatory Conditions

##### Inflammatory Bowel Disease (IBD)

IBD is a heterogenous chronic digestive syndrome characterized by an impaired intestinal barrier and abnormal intestinal repair [65]. This condition can be classified into two different categories, defined as ulcerative colitis (superficial inflammation appears on the colon) and Crohn’s disease (transmural inflammation can occur along the whole gastrointestinal tract). Although the trigger of inflammation is unknown, in subjects with genetic susceptibility, inflammation may be due to exacerbated immune responses to gut microbiota antigens under certain environmental conditions [66]. The release of pro-inflammatory mediators and chronic inflammation has been associated with an increase in noxious bacteria, such as Proteobacteria (e.g., *Escherichia coli*). The increase in pathogenic bacteria can disrupt intestinal permeability, which may impact the composition of gut microbiota, decreasing the variety and abundance of bacteria and leading to intestinal inflammation. In patients with IBD, the abundance of Bacteroidetes and Firmicutes is reduced [67,68]. Moreover, abnormal NAD+ metabolism and increased NAD consumption by NAD-consuming enzymes (PARPs and CD38) have been described in IBD [69], suggesting a link between NAD metabolism and IBD.

##### Systemic Lupus Erythematosus (SLE)

SLE is a systemic autoimmune disease that can affect various organs in the body. While the causes of SLE are not fully known, genetic, environmental, hormonal, and epigenetic factors have been identified as possible contributors [68]. There is evidence of intestinal dysbiosis in both humans with SLE and mouse models of SLE [70]. Furthermore, in individuals with SLE, immune dysfunction can result in a reduced ability to combat bacterial and viral infections [71]. The accumulation of immune complexes in the intestines can trigger inflammatory responses and potentially lead to increased permeability of the gastrointestinal tract, allowing harmful molecules to move from the intestines into the bloodstream [72].

In individuals with SLE with increased disease activity, it has been observed that T cells have higher levels of the CD38 receptor, which acts as a NAD hydrolase, and thereby may contribute to NAD+ depletion in these cells [73]. Furthermore, increased NAD+ consumption in CD8+ T cells has been observed in individuals with SLE, resulting in impaired mitochondrial respiration and reduced cell viability [74]. The impact of altered NAD+ metabolism on various T cell populations indicates that it may have broader implications for the development and progression of SLE [73].

##### Rheumatoid Arthritis (RA)

Both clinical and experimental studies have provided sufficient evidence to support the idea that an ongoing inflammatory response triggered by an imbalance in the gut microbiota can significantly contribute to the onset and progression of RA [75]. Intestinal microbial dysbiosis can affect metabolic function and regulation of the immune response through the gut-joint axis [76]. In addition, the elevated permeability of the gut suggests that gut microbes and their products can migrate to the joint [77].

Recently, it has been found through the analysis of plasma samples that individuals with RA exhibit lower average levels of NAD+ and NADH metabolites than their healthy counterparts [78]. Additionally, a change in NAD+ metabolism has also been detected in RA patients, which is directly linked to the disease’s inflammatory state, and can be reversed with anti-TNF therapy [78]. Furthermore, the potential of using NAD+ enhancers to restore NAD+ levels has been proposed as a potential new clinical strategy to combat inflammation in these patients [78] In this context, treatment with high doses of sustained NAM demonstrated a substantial benefit for RA patients [79,80]; however, its effect on gut permeability was not further studied.

##### Multiple Sclerosis (MS)

Multiple factors have been identified as contributors to the development of multiple sclerosis (MS), including changes in gut microbiota composition, gut-derived substances, intestinal permeability, and dysfunctions in endocrine and enteric nervous systems [81]. MS is characterized by long-term inflammation and damage to the myelin in the central nervous system [82]. In the context of this chronic inflammation, cytokines derived from T helper type 1 cells (Th1 cells) can affect the levels of NAD [82]. Specifically, these cytokines induce the expression of both IDO and CD38, which results in decreased NAD+ levels in microglia and lymphocytes, and thereby contributing to the disease [82]. The impact of MS on intestinal frailty could, at least in part, be due to the nerve damage resulting from NAD+ depletion.

#### 2.2.4. Metabolic-Related Conditions

##### Obesity and Type 2 Diabetes

Obesity is frequently associated with an altered intestinal immunity [83], and deficient insulin signaling further aggravates this complex metabolic context [84]. Intestinal dysbiosis is a frequent feature that links a disturbed intestinal immune system to enhanced metabolic dysfunction [85]. In support of that, an altered intestinal microbiota has been associated with enhanced insulin resistance and development of type 2 diabetes mellitus (T2D) [86], and their main metabolic-related complications, including non-alcoholic fatty liver disease (NAFLD) [87]. Recent accumulating evidence indicates that NAFLD may be closely related to intestinal dysfunction, especially the intestinal microbiota and its metabolites [87].

Intestinal NAD+ content plays an important role in intestinal homeostasis [13]. Interestingly, recent research showed that abrogation of NAMPT, specifically in intestinal epithelial cells, led to reduced local NAD+ levels and impaired intestinal physiology in mice [13] (Table 1). Conceivably, supplementation with a NAD+ precursor (NMN) may result in the restoration of intestinal NAD+ and obesity-related metabolic derangements. Overall, these data, together with those previously described in previous sections, suggest that NAD+ is key in regulating intestinal physiology. However, the impact of NAD+ depletion or its restoration in intestinal inflammation was not assessed in the previous study [13].

Metabolic diseases, such as obesity and diabetes, are frequently characterized by a disturbed NAD+ metabolism [89]. Indeed, decreased NAD+ can predispose to adverse metabolic complications related to obesity and diabetes, including NAFLD [90,91]. Likewise, under such adverse metabolic conditions NAD+ levels may decline and affect many other target tissues and systems. Conceivably, NAD+-increasing strategies may be critical to combat metabolic dysfunction [90,91,92,93].

Intestinal microbiota is altered in obesity [29,94,95], T2D [96], and NAFLD [97]. In the case of T2D, both Bifidobacterium and Bacteroides have been negatively correlated with the presence of diabetes [96]. In addition, the abundance of Lactobacillus has been found to be significantly higher in people with T2D compared to healthy individuals [98]. Gut microbiota directly influences T2D patients’ metabolism via modulating a variety of metabolites from bacterial origin, such as SCFA and bile acids [99]. Likewise, accumulating research also suggests a pivotal role for a disturbed gut-liver axis in the pathogenesis of NAFLD, with gut dysbiosis contributing to this hepatic dysfunction [100,101]. Because NAD+ has been proposed as a potential target to ameliorate NAFLD [102], the potential positive influence of NAD+ precursors on host and microbial physiologies could provide a new perspective for the treatment of metabolic-related diseases.

##### Polycystic Ovarian Syndrome (PCOS)

Currently, the exact pathophysiology of PCOS remains unclear, although various factors, such as genetics, neuroendocrine imbalances, and metabolic disturbances have been proposed as possible causes [103]. Women with PCOS often exhibit metabolic characteristics linked to decreased levels of NAD+, including obesity, insulin resistance, and fatty liver disease [104]. Since PCOS is a chronic inflammatory condition, prolonged and low-level release of proinflammatory factors can lead to persistent damage to the intestinal epithelium and increased intestinal permeability [105]. As a result, several studies have provided evidence to suggest that alterations in the gut microbiota are linked to PCOS; these alterations include a reduction in diversity and changes in the abundance of specific bacterial taxa [106].

##### Cancer Cachexia (CC)

CC is a medical condition that affects patients with advanced cancer, characterized by symptoms, such as anorexia, tissue wasting, and involuntary weight loss, which is related to inflammation, increased gut permeability, and loss of adipose tissue [107,108]. Notably, CC is also associated with a lack of appetite and muscle wasting to gain energy. Interestingly, the same apathy for food is observed in clinical pellagra [4]. In this regard, NAM metabolites detected in the urine are directly related to the worsening of the cachexia state, highlighting the importance of their restoration during CC [109].

The interplay of chronic systemic inflammation, impaired gut barrier function and dysbiosis have been implicated in the etiology of CC [110,111]. NAD+ depletion is a common feature in cancer hosts [112]. NA administration efficiently restored tissue NAD+ and improved the progress of chemotherapy-induced cachexia in different preclinical mouse models of intestinal adenoma/adenocarcinoma. Remarkably, in this same study, muscle *NRK2*, which encodes an NAD+ biosynthetic enzyme [1], was downregulated in cancer patients. In this clinical setting, a decreased expression of *NRK2* was associated with adverse metabolic outcomes in affected individuals, thereby highlighting the meaning of NAD+ in the pathophysiology of CC [112]. In line with this, NR administration has shown promise in preventing and alleviating CC [107]. However, increasing NAD+ could potentially promote tumor growth [113]. Consistently, the downstream target of NAD+, SIRT1, which is responsible for the beneficial effects of NAD+ precursors can exert either pro-carcinogenic or anti-carcinogenic effects depending on the context [114], thereby creating some controversy in the therapeutic use of NAD+-increasing strategies.

#### 2.2.5. Linking Intestinal and Neurological Conditions: Autism Spectrum Disorder

Autism spectrum disorder (ASD) is a neurodevelopmental condition that affects early childhood development, where individuals have difficulties with social interaction, verbal, and non-verbal communication, and exhibit repetitive behavioral patterns [115]. Oxidative stress, mitochondrial dysfunction, and impaired energy metabolism involving NAD+, NADH, ATP, pyruvate, and lactate have been proposed as potential underlying mechanisms of ASD [115]. In children diagnosed with ASD, abnormal NAM-related biomarkers have been detected in both urine and stool samples, suggesting increased NAM breakdown [116]. Furthermore, compared to non-ASD individuals, children with ASD exhibited significantly lower levels of NADH oxidase activity (normalized to citrate synthase activity) in their lymphocyte mitochondria [117].

Remarkably, gastrointestinal issues, such as chronic diarrhea, constipation, irritable bowel syndrome, and gastroesophageal reflux are common comorbidities in ASD, likely arising from a vicious cycle that involves intestinal dysbiosis, intestinal mucosa inflammation and increased intestinal permeability compounded by food selectivity [88]. In this study, elevated levels of urinary nicotinurate and 1-methyl-NAM in ASD children with increased gut permeability revealed disruptions in the tryptophan-NA metabolic pathway, whereas the presence of these catabolites in the urine indicated a shift in tryptophan metabolism from serotonin biosynthesis to the formation of NA [88]. It has been proposed that, in children with ASD, the high level of urinary 1-methyl-nicotinamide could reflect an increased demand for NA [88,118].

## 3. NAD+-Increasing Therapies in the Treatment of Intestinal Inflammation

NAD+ is the central regulator of cellular metabolism [1]. NAD+ and its reduced form, NADH, are used as cofactors in many redox reactions. Moreover, NAD+ acts as a cosubstrate in critical non-redox NAD+-dependent pathways involving PARPs and histone deacetylase sirtuins. There are four different dietary precursors to NAD+: tryptophan, NA, NAM, and NR. Tryptophan can also be derived through the autophagic intracellular proteins recycling to maintain vital NAD+ stores.

**Figure 2:**
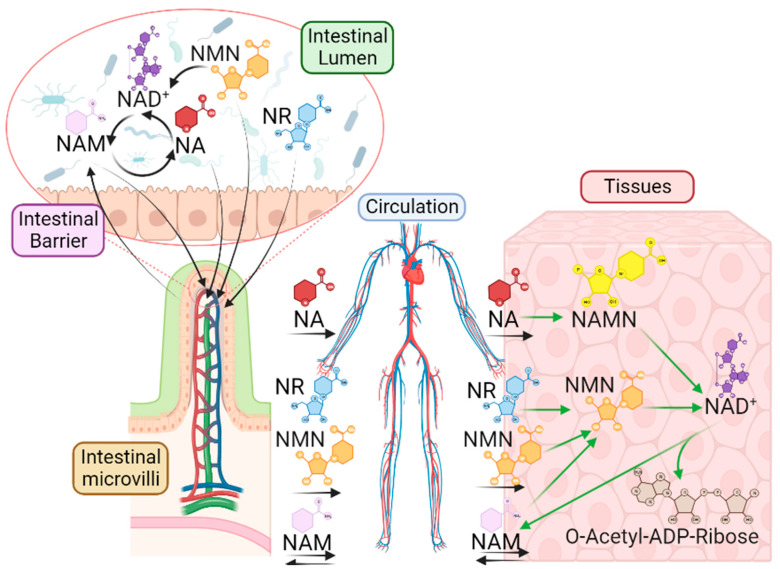
Fate of oral NAD+ precursors. The NAD+ backbone in our organism primarily originates from dietary sources. As such, the metabolic transformation by gut microbiota of oral NMN, NR, and NA, its systemic release and transport to tissues. Within the gastrointestinal tract, NAD+ and NAD+-related molecules undergoes sequential interconversion. NR and NMN can be absorbed and transported in circulation, although NMN can be metabolized to NAM within the gut lumen. NAM can be absorbed or further transformed into NA by gut microbiota. In general, NR, NAM, or NA are the main molecules reaching the portal vein. Subsequently, these metabolites circulate to host tissues where NA can be enzymatically converted into nicotinic acid mononucleotide (NAMN), which can further oxidize to generate NAD+. On the other hand, NMN can be also produced from both NR and NAM, which can then be utilized for NAD+ regeneration. Eventually, NAD+ can be further degraded by various enzyme families, resulting in the production of NAM and O-Acetyl-ADP-Ribose as metabolic byproducts.

As indicated in previous sections, NAD+ deficiency may arise due to inadequate nutrition, adverse metabolic distress, age-related conditions, and immunomodulatory alterations and can be associated with adverse outcomes in different physiological systems, including the intestinal tract. Accordingly, the administration of NAD+ precursors may enhance the NAD+ tissue (Figure 2) content and therefore ameliorate physiopathological outcomes related to NAD+ deficiency.

Given the favorable immunomodulatory and anti-inflammatory properties of NAD+-increasing approaches in different systems [17], increasing NAD+ availability via raising the intake of NAD+ precursors could be considered as a therapeutic strategy to combat intestinal disorders. Most studies have shown that NAD+-increasing therapies have a beneficial effect on intestinal inflammation (Table 2); however, recent findings have been somewhat inconclusive [119,120].

The administration of NAD+ precursors have the potential to exert both direct and indirect beneficial effects against the development of intestinal inflammation. In this regard, the direct effects of NAD+ precursors supplementation include stimulation of GPR109a by using NA, or by inactivation of NF*κ*B via SIRT1, both of which have been reported in previous studies [121]. In addition, recent research has also suggested that some of these molecules may also confer indirect protection against intestinal inflammation by favorably modulating intestinal microbiota [122]. In turn, ingested NAD+ precursors are used by bacteria to produce NAD+, which are also essential for various bacterial metabolic processes. NAD+ can be synthesized through de novo synthesis or from NAD+ precursors [10,123], particularly by bacteria within the Firmicutes and Actinobacteria phyla, which depend on NAD+ precursors for growth [99]. Moreover, NAD+ precursors can modulate intestinal microbiota, and hence influence the synthesis of secondary bile acids and short-chain fatty acids (SCFA), which are relevant for host physiology. Of note, bile acids are produced from cholesterol in the liver and can be secreted into the intestine to promote the absorption of dietary fat and fat-soluble nutrients [124]. Additionally, they can also exert antimicrobial activity by perturbing the bacterial cell membrane, thereby inhibiting bacterial proliferation [124,125,126]. Consistently, the obstruction of bile flow can lead to the proliferation of harmful bacteria in the intestines [126]. Taken together, NAD+-increasing strategies may promote beneficial intestinal bacteria and protect against harmful bacteria by different mechanisms that could act simultaneously.

**Table 2:** In vivo evidence for NAD+-increasing strategies on gut inflammation and dysbiosis in animal models and patients.

Form of B3 Supplement	Disease	Disease Model	Dose and Route Administration	Duration	Benefits in Gut Inflammation/Microbiota	References
NA	Colitis	Wistar rats (adult male 150–200 g) induced by intrarectal administration of iodoacetamide	80 or 320 mg NA/kg was administered orally	Daily for 2 weeks	- Attenuated severity of colitis. - Decreased colonic MPO activity. - Normalized IL-10 level in colon. - Anti-inflammatory changes in a GPR109a-dependent manner.	[127]
NA	Ulcerative colitis	Mutant mice with C57BL/6 genetic background 6- to 8-week-old male mice were administrated with DSS through drinking water (2%) for 6–9 days or pre-sensitized with 1% TNBS at day 1 and then challenged with 2.5% TNBS (100 µL) at day 8.	600 mg NA/kg was administered via gavage	Daily for 6 (DP1) or 9 days (WT)	- Increased PGD2 release in vivo. - Improved colitis by acting on the D prostanoid receptor 1 (DP1). - Promoted clinical remission and mucosal healing in patients with moderately active UC.	[128]
NA	Diarrheal disease	Weaned piglets (Duroc × Landrace × Yorkshire), 21 days old inoculated on the fourth day by oral administration of 4 × 10^9^ cfu/mL enterotoxigenic *E. coli* (ETEC) K88	20 mL nicotinic acid solution with 40 mg NA	Daily for 3 days before *E. coli* inoculation	- Alleviated clinical symptoms, damage to intestinal morphology and inflammation in infected piglets. - Improved the resistance to ETEC K88 infection by enhancing the expression of endogenous antimicrobial peptides. - Boosted the intestinal microbiota diversity and its metabolites. - Reduced the relative abundance of Bacteroidetes, Bacteroidales, and Bacteroidia. - NA regulated histone deacetylase SIRT1 and HDAC7, and specific histone modification sites (acH3K9, acH3K27, and pH3S10) in the promoter region.	[129]
NA	Preventive	Weaned piglets (Duroc × Landrace × Yorkshire), 21 days old, 6.65 ± 0.02 kg body weight	Diet supplemented with 20.4 mg NA/kg	Daily for 14 days	- Promoted intestinal health and reduce inflammation in weaned piglets. - Increased the expression of duodenal transforming growth factor-β, jejunal interleukin-10 and ileal interleukin-6 (IL-6). - Reduced the expression of ileal interleukin-8 (IL-8). - Increased the abundance of Lactobacillus and Dorea, higher levels of butyrate, and decreased the abundance of Peptococcus.	[130]
NAM	Crohn’s disease	Human	600 mg capsules	2 doses daily for 4 weeks	- Potential prophylaxis and therapy for Crohn’s disease.	[122]
NAM	Colitis	C57BL/6J mice (male, >18 weeks old) treated with 1.5% DSS in drinking water for 5 days	30, 60 or 120 mg NAM/kg in drinking water or granules mixed with diet	Daily	- Improvement of disease activity index. - Changes in the relative abundance of Firmicutes and Bacteroidetes. - Decreased MPO content in colonic tissue.	[122]
NAM	Colitis	C57BL/6 WT mice (female, 6–8 weeks 20–28 g) induced by oral infection with *C. rodentium* or by DSS administration	Intraperitoneal injection of 250 mg/kg from day two before infection (preventive) or two days post infection (therapeutic) until the end of experiment	Daily for 12 days	- Decreased systemic bacterial invasion. - Decreased histological damage. - Increased fecal clearance of *C. rodentium* by up to 600-fold. - Influenced the composition of the fecal microbiota.	[131]
NAM	Mild colitis	C57BL/6 mice Ace2-/y (male) with DSS	Trp1 diet and 0.4 g/L NAM in drinking water	Daily for 13 days	- Alleviation of severe colitis and diarrhea. - Favorable influence in the composition of intestinal microbiota in treated Ace2 mutant mice. - Key regulation of gut microbiota and attenuation of intestinal inflammation.	[132]
NMN	None	C57BL/6J mice (female, 12-week-old)	0.1 mg/mL, 0.2 mg/mL, 0.4 mg/mL, and 0.6 mg/mL in drinking water	15 weeks	- Increase in probiotics and decrease in several harmful bacteria. - Long-term NMN treatment reduced intestinal mucosal permeability and exerts a protective effect on the intestinal tract.	[18]
NMN	Colitis	C57BL/6J mice (male, 8 weeks old) treated with drinking water containing 3% dextran sodium sulphate (DSS)	1 mg NMN/g was administered via gavage	Daily for 3 weeks	- Improved morphology of inflamed intestines. - Mildly restored length of colon improved barrier function. - Reduced abundance of serum proinflammatory factors. - Favorable changes in the composition and abundance of intestinal microbiota.	[19]
β-NMN	Peritonitis	C57BL6 mice (male, 7–12 week-old) with cecal ligation and puncture (CLP)	Intraperitoneal injection at 185 mg β-NMN/kg, 4 days after CLP and for 3 days post thioglycolate treatment	Daily for 4 days	- Downregulated genes controlling the immuno-inflammatory response. - Abrogation of the pro-inflammatory M1 macrophages and induction of specific markers of M2 macrophages. - Reduction in phagolysosome acidification and secretion of inflammatory mediators in macrophages.	[133]
NR	None	C57BL/6N mice (male, 8–10-week)	400 mg NR/kg by oral administration	Single	- Diphasic replenishment of NAD+. - Gut microbiota contributed to the NAD+ synthesis from NR.	[134]
NR+ NRTBCl NRTOCl	None	Gallus gallus	Administration of 30 mg/mL NRCl, NRTBCl or NRTOCl via intra-amniotic	Day 17 of embryonic incubation	- Effects on the composition and function of cecal microbial populations (i.e., showing NR-mediated elevations of the relative content of Clostridium; NRCl-mediated increase in populations of Bifidobacterium, Lactobacillus, and *E. coli*). - NRCl, NRTBCl, and NRTOCl upregulated the expression of ZnT1, MUC2, and IL6. - NRCl and its derivatives increased expression of brush border membrane digestive proteins.	[135]

Abbreviations used: DSS, destran sulfate sodium, MPO, myeloperoxidase, NA, nicotinic acid, NAM, nicotinamide, NMN, nicotinamide mononucleotide, NR, nicotinamide riboside, NRTBCl, NR+ tributyrate chloride, NRTOCl, NR+ trioxide chloride, NRCl, NR+ chloride, TNBS, trinitrobenzene sulfonic acid.

### 3.1. Nicotinic Acid (NA)

In the early 20th century, NA was recognized as a potent therapy for pellagra [6]. At pharmacological doses, NA is known to act as a broad-spectrum lipid-modulating agent [136]. Although the molecular mechanisms responsible for its effects on lipid metabolism have been identified, the molecular mechanism underlying its anti-inflammatory effects through its binding to GPR109a was elucidated more recently [8,137].

Emerging evidence supports the potential of NA as an anti-inflammatory therapy to combat inflammation-associated metabolic disorders, including intestinal inflammation. In support of this notion, reduced levels of NA have been detected in the feces of IBD patients compared with non-IBD subjects [138]. Moreover, some in vitro studies have demonstrated that NA significantly reduced the production of IL-8 in both LPS and IL-1β stimulated Caco-2 cells, as well as restored the levels of several metabolites that were modified by the inflammatory stimulus. These findings also confirm the anti-inflammatory effect of NA on intestinal inflammation and demonstrate the possible use of NA as an anti-inflammatory compound. Therefore, NA may be considered as a promising starting point for further investigation of its beneficial effect in IBD [139].

Recent studies have revealed that the presence of adherent-invasive *E. coli* (AIEC) in the intestinal microbiota [140] is linked to the beneficial effects of NA against gastrointestinal diseases, such as IBDs and inflammation-associated colorectal cancer. These AIEC can adhere to and invade epithelial cells and survive and replicate within resident macrophages. Recent nutrient supplementation studies have identified a specific strain of AIEC (NC101) that is auxotrophic for NA due to a missense mutation in *nadA*, a gene which encodes quinolinate synthase A, an important enzyme for de novo NAD+ biosynthesis. The NA auxotrophy confers pro-carcinogenic activity to this specific strain of *E. coli*, but not in the other non-toxigenic *E. coli* or other AIEC strains.

In the context of intestinal inflammation, NA treatment has been reported to provide protection against experimental colitis in mice (Table 1) [128] and rats [127]. In one of these studies, NA administration ameliorated vascular permeability, prevented apoptosis of epithelial cells, and attenuated the pro-inflammatory gene expression in resident macrophages [128]. Notably, the NA-mediated protection against induced colitis was found to be via prostaglandin D2, in a D prostanoid receptor 1 (DP1)-dependent manner [128]. On the other hand, NA has shown a favorable influence on the severity of colitis as revealed by a concomitant normalization of the colonic levels of myeloperoxidase (MPO) activity, a microbial biomarker, and TNF-α and IL-10 levels [127]. In this set of experiments, the GPR109a receptor-dependent effect of NA was elegantly demonstrated by using mepenzolate bromide, a GPR109a receptor blocker. Indeed, this drug abolished the NA-mediated amelioration in MPO and other metrics of colitis, thus inferring that the favorable NA-mediated effects on pathologic angiogenesis and inflammatory changes were via the GPR109a receptor [127].

The protective contribution of NA on growth performance and gut health has also been shown in weaned piglets [130] (Table 2). Weaning is a highly stressful event in the life of piglets that often results in intestinal and immune distress [141,142,143] that can lead to severe diarrhea [144]. Therefore, prevention of impaired intestinal barrier function and inflammation induced by weaning stress may be a potential strategy. Despite NA being a common additive in pig diets, the underlying mechanism remains unclear, as it has been shown to reduce pig health, growth, and feed intake, particularly during the first week after weaning.

NA supplementation in weaned piglets resulted in a significant reduction of the inflammatory response in the intestinal mucosa as compared with control piglets and those given an NA receptor antagonist [130]. Remarkably, this NA-mediated effect was accompanied by an improvement in the relative abundance of beneficial bacteria in the colon, indicating a potential role for NA in modulating the gut microbiota [130]. In another independent study using weaned piglets, NA significantly alleviated the intestinal morphological damage caused by enterotoxigenic *E. coli* K88 infection [129]. Importantly, this effect was followed by a significant reduction in the expression of different pro-inflammatory cytokines in both the serum and intestines of treated weaned piglets. Moreover, NA supplementation also leads to an increase in the levels of IgA and the expression of antimicrobial peptides in the intestinal tract of treated weaned pigs, as well as changes in the activity of histone deacetylases SIRT1 and HDAC7 and concomitant differential acetylation patterns in different targets (i.e., acH3K9, and acH3K27) [129]. Additionally, NA administration positively influenced changes in the intestinal microbiota, as revealed by reductions in the relative abundance of the phyla Bacteroidetes, Bacteroidales, and Bacteroidia, and their metabolites. Another study, also conducted in weaned piglets [145], reported that NA supplementation positively influenced different antioxidant parameters, such as malonyldialdehide, and different forms of the enzyme activity superoxide dismutase, i.e., T-SOD, and CuZn-SOD. Moreover, NA supplementation improved the inflammatory status by promoting the downregulation of TNF-α and COX2 in the jejunal mucosa. These changes were linked to significant differences in the composition of the colonic species, which were also accompanied by changes in the isovaleric acid content. Overall, studies evaluating NA as an intervention have provided new insights into the beneficial effects of NA on gut physiology and microbiota.

### 3.2. Nicotinamide (NAM)

The administration of NAM has also been proven effective in modulating intestinal inflammation [122] (Table 2). Studies have revealed that Crohn’s disease activity index in affected patients was improved when treated with high-dose NAM for 4 weeks. Likewise, treatment with NAM ameliorated the disease activity index and MPO content in colonic tissue of mice with dextran sodium sulfate (DSS)-induced colitis. Interestingly, such amelioration was accompanied by changes in the intestinal microbiota in treated mice. Similarly, in a recent study, NAM supplementation in C57BL/6 male mice by administration of 1.5% DSS showed restoration of the loss of alpha and beta diversity and increased richness of the gut microbiota. In addition, NAM also modulated specific bacteria, including Odoribacter, Flexispira, and Bifidobacteria, in the group of mice with chronic colitis that were treated with NAM. In this same study, the phylogenetic investigation of communities by reconstruction of unobserved states (PICRUSt) analysis indicated that NAM treatment restored impaired functions of the gut microbiota (replication and repair, and cell motility) in mice with DSS-induced colitis. Furthermore, NAM also restored the reduction in valeric acid in mice with DSS-induced chronic colitis. Consistent with another report [21], fecal SCFA levels did not differ among groups, except for valeric acid, which was restored after NAM treatment. Similar results were reported in another mouse model of mild colitis [132]. Overall, the data suggested that NAM could alleviate DSS-induced chronic colitis in mice by inhibiting inflammation and regulating the composition and function of gut microbiota, highlighting its therapeutic potential.

In another independent study, NAM supplementation in C57BL/6 mice with bacterial (oral infection with *Citrobacter rodentium*) or chemical-induced colitis (DSS administration) increased the fecal excretion of *C. rodentium* and enhanced expression of antimicrobial peptides in neutrophils, in parallel to the alleviation of colon histological damage, and reduction in invasive *C. rodentium* infections. Additionally, in parallel to these favorable changes, NAM supplementation also lowered the abundance of representatives of the phylum Firmicutes, which are frequently linked to obesity and metabolic disorders [131]. Overall, these findings suggest that NAM could be a promising therapeutic agent for the prevention and treatment of bacterial infections and associated intestinal disorders.

### 3.3. Nicotinamide Mononucleotide (NMN)

NMN is a precursor of NAD+ and has been shown to modulate the gut microbiota and inflammation in different dietary and pharmacological mouse models of impaired gastrointestinal barrier function. Long-term administration of NMN was shown to result in a rise in the levels of beneficial gut bacteria, such as Lactobacillus, Bifidobacterium, *Ruminococcae*_UCG-014, *Prevotellaceae*_NK3B31_group, and *Akkermansia muciniphila* [18]. The latter is a gut commensal bacterium that has been associated with metabolic health and a reduced risk of obesity and diabetes, and which is known to produce butyric acid and have probiotic properties [146]. In contrast, the presence of harmful bacteria, such as Bilophila, Desulfovibrio, *E. coli* and Oscillibacter decreased significantly in the intestinal microbiota of mice. This shift in gut microbiota composition was associated with improved intestinal barrier function, reduced inflammation, and increased mucus secretion in the gut. Furthermore, NMN supplementation also improved the expression of genes involved in maintaining intestinal homeostasis and prevented the disruption of intestinal barrier function caused by a high-fat diet [18].

In addition, the administration of NMN has shown potential in improving the morphology and barrier function of inflamed intestines and reducing the expression of proinflammatory factors in the serum of mice with induced experimental colitis [19]. A similar anti-inflammatory effect was also observed in a mouse model of peritonitis [133]. Moreover, NMN promoted significant changes in the composition and abundance of intestinal microbiota in this mouse model of IBD. Indeed, NMN significantly increased the relative fecal abundance of Firmicutes, Verrucomicrobia, Akkermansia and Lactobacillus, considered as beneficial bacteria, which are involved in the production of SCFAs (acetic, propanoic and butyric acids). In addition, members of Bacteroidetes phylum and *Muribaculaceae* unclassifiable were decreased. While NMN could not completely rescue intestinal inflammation, its supplementation could potentially prevent the development of disease and relieve pain induced by IBD [19].

In line with this, the supplementation of aged mice with NMN significantly increased the jejunal NAD+ content, and improved the jejunal structure, while having little impact on the colonic microbiota and NAD+ content [147]. These findings were accompanied by a favorable upregulation of genes involved in the antioxidant response (i.e., nuclear factor E2-related factor 2, heme oxygenase-1, and superoxide dismutase 2), and the gastrointestinal barrier stability (i.e., occludin, and claudin-1). In addition, NMN administration downregulated the expression of tumor necrosis factor alpha (TNF-α). Interestingly, the addition of NMN to D-galactose (D-gal)-induced senescent IPEC-J2 cells normalized the expression of proinflammatory targets and improved their antioxidant capacity [147].

NMN supplementation has also been found to be effective in alternative contexts related to disturbed intestinal homeostasis. For instance, in radiation-induced gastrointestinal damage, NMN alleviated radiation-induced intestinal fibrosis in C57BL/6J mice after receiving a 15 Gy dosage of abdominal irradiation. The long-term NMN-related attenuation of radiation-induced intestinal fibrosis was accompanied by concomitant favorable changes in the composition of gut microbiota [148]. Consistent with this view, NMN has also been shown to ameliorate the intestinal functional disturbances and dysbiosis linked to other intestinal distress situations, such as that induced by sleep deprivation in mice [149]. Interestingly, NMN not only improved intestinal physiology and microbiota, but also restored colonization resistance against intestinal infections.

### 3.4. Nicotinamide Riboside (NR)

NR administration also influences intestinal microbiota [21,134,135]; however, its effects appear to be species-specific [21]. A study found that dietary supplementation of NR induced changes in the composition of gut microbiota in both rats and mice, favoring bacterial species capable of synthesizing NAD+ from specific precursors. However, no such change was seen in humans. In rats, dietary NR increased the relative abundance of species in the *Erysipelotrichaceae* and *Ruminococcaceae* families within the Firmicutes phylum in feces; in particular, the PnuC-positive bacterial strains, which express the NR-specific transporter, showed an increased growth rate [123,150]. Moreover, bacterial metabolization of NR positively influenced the pool of NAD+ metabolites available for the host, and to produce other metabolites directly sensitive to changes in the intestinal microbiota, such as secondary bile acids and SCFAs. Although intestinal inflammation was not evaluated in this study, SCFAs, such as butyrate, attenuates intestinal inflammation, thereby maintaining intestinal barrier integrity and motility [151].

In this same study, NR intake in mice also influenced the gut microbiota after 12 weeks of treatment, but the observed changes differ from those seen in rat feces [21]. In mice, NR intake increased the relative proportion of *Lachnospiraceae* (Firmicutes phylum) species. Species belonging to the *Lachnospiraceae* family (i.e., *Eubacterium halli*) have been reported to be important butyrate producers found in the gut microbiota [151].

In contrast to rats and mice, in humans, NR did not modulate the alpha and beta diversity and bacterial composition of fecal microbiota and was unlikely to influence bacterial species capable of synthesizing NAD+ from specific precursors [21]. Nonetheless, some benefits on intestinal inflammation could be inferred from favorable changes in fecal composition of microbiota, and therefore future studies evaluating the therapeutic potential of NR in humans may be required.

## 4. Conclusions and Perspectives

Gut homeostasis is often disturbed in diverse physiopathological conditions and may contribute to altered host physiology. Accumulating experimental and clinical evidence links NAD+ deficiency to inflammation in several immunomodulatory and metabolic conditions that are frequently associated with disturbed gut homeostasis. Importantly, recent research also suggests a role for different NAD+ precursors as possible regulators of gut physiology and their therapeutic potential to treat or ameliorate intestinal inflammatory complications. Indeed, NAD+ precursors have been shown to positively modulate gut inflammation through different mechanisms. As such, NAD+-increasing strategies could be considered as a potential therapeutic approach. In this context, accumulating data suggest that NAD+ precursors could, at least in part, improve gut dysfunction through favorably modulating intestinal microbiota. Intestinal microbiota can convert host NAM into NA, which is used for NAD synthesis in host tissues (Figure 2). Likewise, both oral NR and NMN can also contribute to host NAD+ via its conversion into NA by the intestinal microbiota. However, the efficacy of different NAD+-raising approaches to combat intestinal disorders under different immunomodulatory/metabolic states of the host are topics that remain to be further researched. The bidirectional influence of host micronutrients on the intestinal microbiome is still under debate, as they can serve as a food source for the microbiome while also being transformed into other molecules that enhance host metabolic flexibility.

## Figures and Tables

**Table 1:** NAD+ deficiency and intestinal inflammation in different pathophysiological settings ^†^.

Disease	Characterization in Humans or Disease Model	NAD Deficiency or Metabolic Alteration	References
Pellagra	Human	Primary pellagra is characterized by a cellular deficiency of NAD+ caused by insufficient intake of dietary precursors	[6,22]
Alcoholism	Human	Secondary pellagra as a result of malnutrition, which is also linked to NAD+ deficiency	[6]
Chemotherapy	Human	Chemotherapy can induce pellagra symptoms due to excess NAD+ consumption	[6,22]
Inflammatory bowel disease	Human	Increased cellular NAD consumption in IBD.	[23,67]
Autism spectrum disorder	Human	Irritable bowel syndrome, and gastroesophageal reflux are common comorbidities in ASD; increased gut permeability related to disruptions in the tryptophan-NA metabolic pathway	[88]
Obesity	Mouse model	Abrogation of NAMPT specifically in intestinal epithelial cells led to reduced local NAD+ levels and impaired intestinal physiology.	[13]

^†^ Only evidence-based data linking NAD+ deficiency and intestinal inflammation were shown in both experimental and clinical scenarios. Abbreviations used: ASD, autism spectrum disorder; ATP, adenosine triphosphate; LPS, lipopolysaccharide; NAD+, oxidized form nicotinamide adenine dinucleotide; ROS, reactive oxygen species.
